# NMDA Receptor Antagonist Attenuates Bleomycin-Induced Acute Lung Injury

**DOI:** 10.1371/journal.pone.0125873

**Published:** 2015-05-05

**Authors:** Yang Li, Yong Liu, XiangPing Peng, Wei Liu, FeiYan Zhao, DanDan Feng, JianZhong Han, YanHong Huang, SiWei Luo, Lian Li, Shao Jie Yue, QingMei Cheng, XiaoTing Huang, ZiQiang Luo

**Affiliations:** 1 Department of Physiology, Xiangya School of Medicine, Central South University, Changsha, Hunan, China; 2 Department of Pediatrics, Xiangya Hospital, Central South University, Changsha, Hunan, China; 3 Xiangya Nursing School, Central South University, Changsha, Hunan, China; 4 Xiangya School of Medicine, Central South University, Changsha, Hunan, China; French National Centre for Scientific Research, FRANCE

## Abstract

**Background:**

Glutamate is a major neurotransmitter in the central nervous system (CNS). Large amount of glutamate can overstimulate N-methyl-D-aspartate receptor (NMDAR), causing neuronal injury and death. Recently, NMDAR has been reported to be found in the lungs. The aim of this study is to examine the effects of memantine, a NMDAR channel blocker, on bleomycin-induced lung injury mice.

**Methods:**

C57BL/6 mice were intratracheally injected with bleomycin (BLM) to induce lung injury. Mice were randomized to receive saline, memantine (Me), BLM, BLM plus Me. Lungs and BALF were harvested on day 3 or 7 for further evaluation.

**Results:**

BLM caused leukocyte infiltration, pulmonary edema and increase in cytokines, and imposed significant oxidative stress (MDA as a marker) in lungs. Memantine significantly mitigated the oxidative stress, lung inflammatory response and acute lung injury caused by BLM. Moreover, activation of NMDAR enhances CD11b expression on neutrophils.

**Conclusions:**

Memantine mitigates oxidative stress, lung inflammatory response and acute lung injury in BLM challenged mice.

## Introduction

The development and progression of many acute and chronic lung disorders are associated with excessive or unresolved inflammation, which can result in cell injury and other pathological consequences. Bleomycin (BLM) is widely used to induce acute lung injury (ALI) and fibrosis in murine models. Intranasal administration of BLM leads to the early stage of inflammatory response and the late stage of collagen deposition. The pathological alterations include injuries of alveolar epithelial cells (AECs) and vascular endothelial cells (VECs), alveolar neutrophilic recruitment, and up-regulation of pro-inflammatory cytokines [1–3]. Furthermore, it is well known that activated and accumulated inflammatory cells in the lungs release toxic reactive oxygen species (ROS) that leading to lung injury [4].

Glutamate (Glu) is the main excitatory neurotransmitter in the central nervous system (CNS). Under pathological conditions, extracellular glutamate concentrations are increased by abnormal release and/or clearance. This causes overstimulation of glutamate receptors, resulting in neuronal injury or death, known as excitotoxicity [5]. Glutamate neurotoxicity plays an important role in many neurological disorders [6]. The functions of glutamate and its receptors have been well-characterized in the central nervous system. N-methyl-D-aspartate (NMDA) receptors (NMDARs) are the principal receptors in mediating Glu neurotoxicity [7]. It has been reported that NMDAR presents in non-neuronal tissues and cells, including kidney, lung, urogenital tract, pancreatic β cells, and blood vessels [8–11]. Functional NMDARs are expressed on mononuclear leukocytes, neutrophils and alveolar macrophages [12–14]. NMDAR activation leads to increased recruitment of mononuclear leukocytes, neutrophils and macrophage in retina and striatum [15], and up-regulation of neutrophils activation [16]. Similar to neurons, mononuclear leukocytes and neutrophils can release glutamate, which can further exacerbate blood brain-barrier-injury [12, 13]. Several lines of evidence indicate that NMDARs play an important role in regulating inflammation in neuronal and non-neuronal cells and tissues, such as chronic morphine-induced neuroinflammation, retinal damage, arthritis and cardiac inflammation [15, 17–19]. Activation of NMDA receptors can induce acute high-permeability edema in isolated rat lungs [20]. Our previous work also showed that Glu (0.5g/kg, ip) in vivo provoked acute lung injury [21] and NMDAR antagonist MK-801 attenuated hyperoxia induced lung injury [22].

BLM, a chemotherapeutic drug used clinically for treatment of a variety of human malignancies, has been shown to induce, at the high doses, lung injury and pulmonary fibrosis in patients [23]. Therefore, BLM is used widely as an agent to induce experimental lung fibrosis in rodents [24]. Intratracheally administration of BLM causes acute lung inflammation during the first week and pulmonary fibrosis in the second and third week post BLM [25]. It was demonstrated that treatment with dexamethasone in the first three days after BLM challenge prevented the development of BLM induced fibrosis [26]. This indicates that the acute inflammation reaction plays a major role in the development of pulmonary fibrosis induced by BLM. Although there have been reported that, NMDARs play an important role in allergic, heat, LPS and hyperoxia-induced acute lung injury [22, 27–30], the role of NMDARs in BLM induced-lung injury remains unclear. In order to investigate the mechanism of BLM-induced lung injury, we hypothesize that activation of NMDAR mediates BLM-induced acute lung injury, and that blocking NMDAR could attenuate lung injury. Our results showed that NMDAR antagonist memantine attenuated BLM-induced early inflammation and suggested that memantine may protect lungs from BLM-induced fibrosis.

## Materials and Methods

### Ethics statement

The Ethics Committee of Institute of Clinical Pharmacology at Central South University (Changsha, China) approved the experiments, which were performed in accordance with the guidelines of National Institutes of Health. Before surgeries, mice were anesthetized with chloral hydrate (400mg/kg given i.p.), and necessary efforts were taken to minimize suffering.

### Animal model and experimental design

Female C57BL/6 mice, weighting 18–20 g, were purchased from JingDa Laboratory Animal Company (Changsha, China) and were maintained in 12-hour light, 12-hour dark cycles with free access to food and water in accordance with guidelines from the Committee on Research Animal Welfare of Central South University, Changsha, China. Mice were randomly divided into four groups: (1)intratracheal saline plus intraperitoneal saline (Con); (2)intratracheal saline plus memantine (Me, 10mg/kg/day); (3)intratracheal bleomycine (BLM, 5mg/kg) plus intraperitoneal saline (BLM); (4)intratracheal BLM plus memantine (BLM+Me). After being anesthetized, mice were intratracheally injected with 5mg/kg bleomycin (Tokyo, Japan) in 50 μl saline on Day 0. Memantine (Sigma, St. Louis, MO) was administered intraperitoneally every day from Day 0. At 3 or 7days, animals were sacrificed under anesthesia and exsanguination. BALF and lung tissues were collected for the following assays. Total 320 mice were used in experiments.

### Histological analysis

The left lobe from non-lavaged lung was fixed with 4% paraformaldehyde in phosphate-buffered saline for 24 h and then embedded in paraffin. Five-micron thick sections were stained with Hematoxylin Eosin (HE). Alveolitis was determined with the H&E stained sections according to previously published criteria [31]. Briefly, inflammatory infiltration and the area of involved lesions of lung sections were graded: grade 0, normal tissue; grade 1, (<20% of the slide), 2(20%-50% of the slide) and 3(>50% of the slide). The mean score from all examined fields was calculated as the inflammation score (IS).

### Wet/Dry weight ratio assay

The lungs were collected and weighed before and after being dried in the incubator at 60C, for 72 h.

### Bronchoalveolar lavage

The mice were anesthetized and a plastic cannula was inserted into the trachea. Lavage was performed with a 0.8 ml of 0.9% saline solution. This procedure was repeated three times. The bronchoalveolar lavage fluid (BALF) samples were centrifuged, and the cell pellets were resuspended in PBS and the supernatant were stored at -80°C for further research.

### Glutamate concentration in the BALF

The concentration of glutamate in BALF from Day 3 was measured by OPA-FMOC-CL precolumn derivatization high performance liquid chromatography (HPLC) (G4212A, Agilent 1290, U.S.A.) using ultraviolet detection. Two microliters of BALF, 5μl Borate saline buffer were mixed and reacted for 0.2min at room temperature. Then a 1μl OPA solution and a 1μl FMOC-CL solution were added into the mixture and mixed. Then a 4μl aliquot of the reaction mixture was injected into the HPLC system. The separation was performed on a Aglient Zorbax AAA column (5μm, 4.6×150mm) with the mobile phase of 20mmol•L-1sodium acetate buffer (PH 7.2), 100μl Triethylamine and 2.5ml tetrahydrofuran (phase A) and a mixture of V:V:V = 1:2:2, 20mmol•L-1sodium acetate buffer(PH 7.2), Acetonitrile and Methyl alcohol (phase B). The analysis was performed with a linear gradient from A:B (99.5:0.5) to 45% B within 25min; 100% B within 26.1min; then eluted with 100% B for 30min to elute other components. The flow rate was set to 200μl/min. The signal A and signal B wave lengths were 338nm and 262nm, respectively.

The concentration of glutamate in BALF of Day 7 was measured using a commercially available kit (Nanjing Jiancheng Bioengineering Institute, Nanjing, China).

### Real-time PCR

Total RNA was extracted from whole lung with Trizol reagent (Invitrogen, USA), and the synthesis of the cDNA was performed with random hexamers. The primers were used as following, IL-1β (forward primer, 5’-GCCCATCCTCTGTGACTCAT-3’, reverse primer, 5’-AGGCCACAGGTATTTTGTCG-3’), β-actin(forward primer,5’- GGCTGTATTCCCCTCCAT-3’, reverse primer,5’-CCAGTTGGTAACAATGCCATGT-3’), TNFα (forward primer, 5’- ACAGCAAGGGACTAGCCAGGAG-3’, reverse primer, 5’- GGAGTGCCTCTTCTGCCAGTTC-3’), IL-10 (forward primer, 5’-GGGTTGCCAAGCCTTATCGGAA-3’, reverse primer, 5’-CTGCTCCACTGCCTTGCTCTTA-3’). A SYBR Green quantitative PCR was done on Bio-Rad real-time PCR system. Each experiment was performed in duplicate and repeated three times.

### Preparation of lung tissue for biochemical analyses

Oxidative stress was evaluated by detecting lung homogenate levels of malondialdehyde (MDA). The MDA concentrations were determined by measurement of thiobarbituric acid (TBA) reactivity according to commercially available kit (Nanjing Jiancheng Bioengineering Institute, Nanjing, China). TBA was added into lung homogenate, and the mixture was centrifuged. The supernatant was obtained then measured at 532nm with a spectrophotometer. Neutrophil accumulation was determined by myeloperoxidase (MPO) activity of lung homogenate. The assay of MDA and MPO were performed following the instructions of the detection kits (Jiancheng Biotech, Nanjing, China).

### Pulmonary vascular permeability

Mice were sacrificed on Day 3. Evans blue dye (20 mg/kg) in 250 μl of 0.9% saline was infused into tail vein and allowed to circulate for 60 min. BALF were collected and centrifuged at 14,000 ×g revolution/min for 20 min at 4°C, then the supernatant was removed to measure the optical density at 620 nm.

### ELISA

An enzyme-linked immunosorbent assay (ELISA) was used to detect concentrations of the cytokines tumor necrosis factor alpha (TNFα), interleukin-1 beta (IL-1β) and interleukin-10 (IL-10) in lungs. The lungs were homogenized in phosphate-buffered saline (PBS, pH 7.4), and centrifuged at 10,000×g to removed insoluble debris. The supernatants were assayed with ELISA kit according to the manufacturer’s instructions (CUSABIO, China).

### CD11b expression on neutrophils

NMDA was added into 100 μl anticoagulated whole peripheral blood to a final concentration of 10^-6^ M, and then the mixture was further incubated for 4 h. Fluorescein isothiocyanate (FITC)-conjugated mouse anti-human CD11b antibody (Biolegend) was added and the mixture was incubated on ice for 30 min. Neutrophils were obtained from the whole peripheral blood and the cells were washed twice in PBS containing 0.2% BSA. The histograms were acquired on a FACS Calibur instrument using CELLQUEST software (Becton Dickinson).

### Statistics

Comparisons were made by using one-way analysis of variance followed by the Student-Newman-Keuls (SNK) test for multiple comparisons or the nonparametric test (Mann-Whitney U test), depending on the distribution of the data. P<0.05 was considered statistical significant.

## Results

### Level of Glu in BALF after BLM challenge

**Fig 1:**
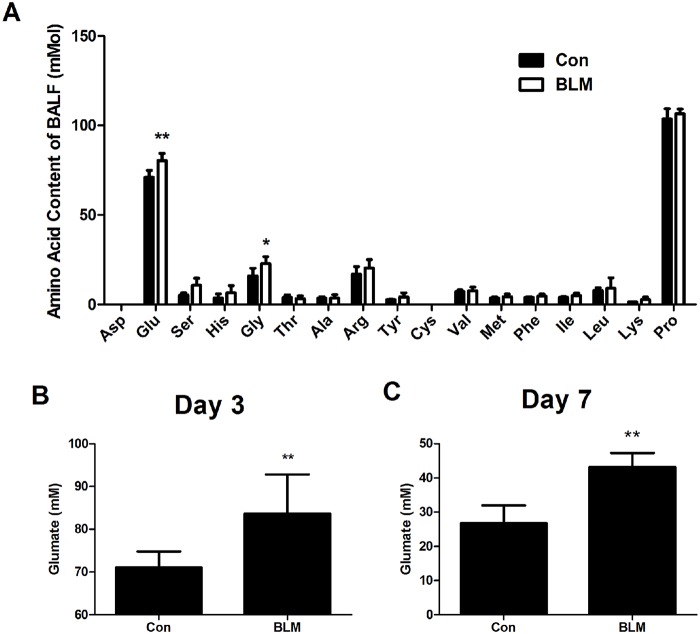
Concentrations of 17 kinds of amino acid and glutamate in BALF after BLM-induced injury. Lungs were lavaged and BALF was analyzed for 17 kinds of amino acid concentrations (A) and glutamate concentration at 3days (B) and 7days (C) after BLM instilled. n = 5–10. Bars: mean ±SD. **P<0.01 vs Con group. The experiment was repeated three times in the same condition.

In order to check if Glu was released after BLM challenge in lungs at Day 3, we used HPLC to determine the changes of 17 kinds of amino acid in BALF. Data showed that, three days after BLM injured, only the concentration of Glu and Gly in BALF were higher in the BLM group than in the saline control group(Fig 1B, P<0.01). The data for the other 15 kinds of amino acid except Asp and Cys which weren’t detected because their content were lower than their standard samples, showed that the concentration of Ser, His, Thr, Ala, Arg, Tyr, Val, Met, Phe, Ile, Leu, Lys and Pro had no difference between saline control group and BLM group (Fig 1A, P>0.05). In addition, the concentration of Glu in BALF of Day 7 was significantly higher in BLM group than that the Con group (Fig 1C, P<0.01). This indicates that endogenous Glu and Gly were selectively released from lungs at the beginning of BLM-induced acute lung injury.

### Effects of memantine on acute lung inflammation induced by BLM

**Fig 2:**
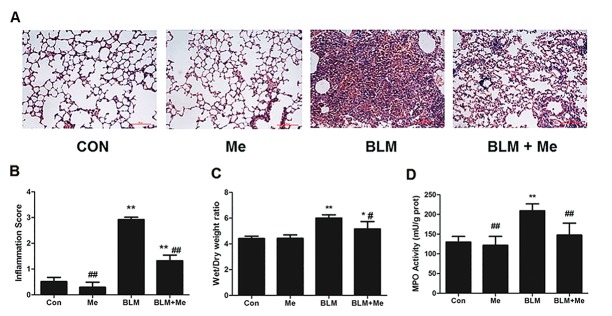
Memantine reduces bleomycin-induced alveolitis and neutrophil infiltration. The lung tissues were collected on Day 7. (A) The histopathologic examination was determined by H&E staining. (×200, Scale bar = 100 μm). (B) The comparisons of the inflammation score values in each group. n = 4–7. (C) W/D ratio, n = 5–9. (D) The activity of MPO in lung homogenate, n = 3–6. Bars: mean ±SD. *P<0.05, **P<0.01 vs control group. ^#^P<0.05, ^##^P<0.01 vs BLM group.

To investigate the target of released Glu in BLM injured lung, we observed the effect of memantine, an NMDAR antagonist, on acute inflammation induced by BLM. Fig 2A is the representative H&E stained lung sections. Upon BLM injection, we observed obvious lung inflammatory response, including significant interstitial infiltration of inflammatory cells and thickening of the alveolar walls (Fig 2A) on Day 7. Moreover, BLM-induced lung histological alterations were significantly attenuated by memantine treatment. Normal lung structure was not changed by memantine treatment alone. As we showed in Fig 2B, the magnitude of inflammation (presented as inflammation score) of the BLM group was significantly higher than that of control group on day 7, which was significantly reduced by memantine treatment. In addition, compared to the BLM-treated mice without memantine, the mice treated with memantine showed a significant reduction in the lung W/D ratio (Fig 2C). MPO activity, an indirect index of neutrophil infiltration in lung tissue that was significantly increased on Day 7 in BLM group as compared with control group. Memantine treatment inhibited BLM-induced MPO activity increase (Fig 2D). These results showed that memantine can attenuate BLM-induced acute lung injury, which indicated that the release of endogenous Glu induced acute lung inflammation by activation of NMDAR.

### Effects of Memantine on Cytokines expression in lung homogenate

**Fig 3:**
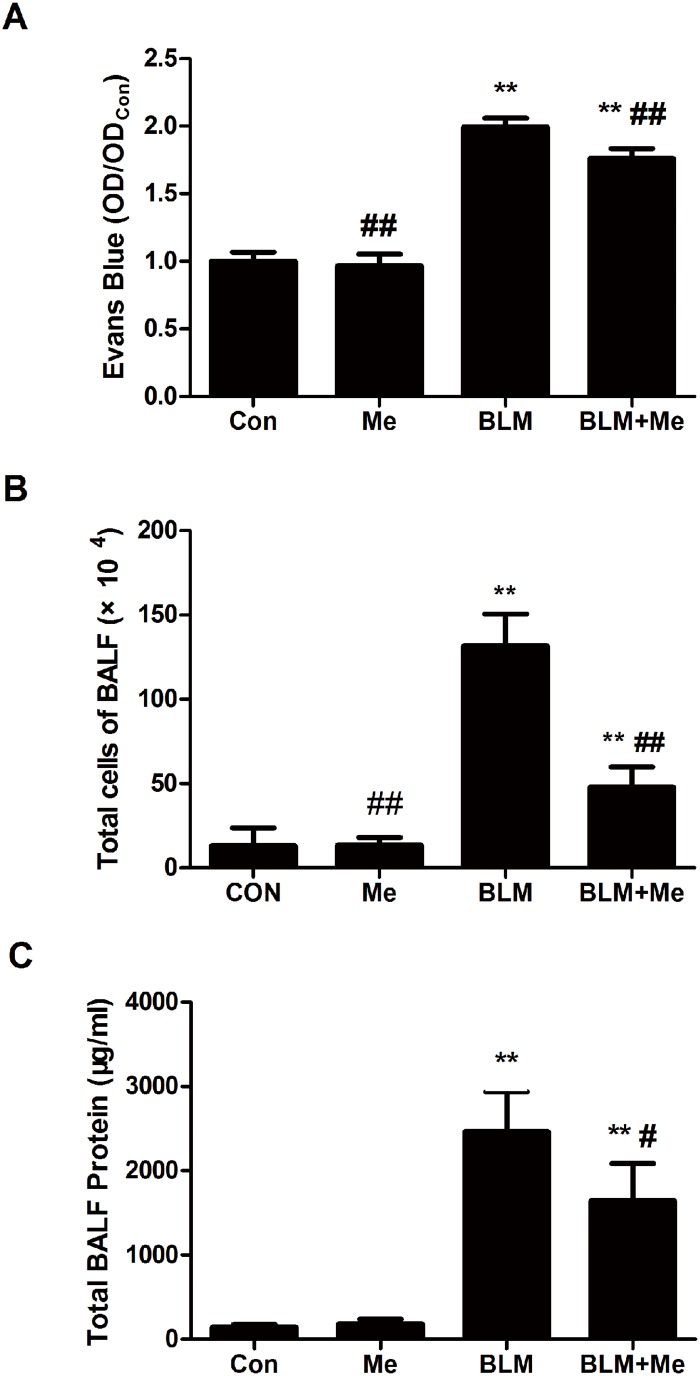
Memantine reduces pulmonary vascular permeability and inflammatory cell infiltration. Lungs were lavaged and BALF was analyzed for Evans Blue, n = 4 (A), total cells, n = 5–6 (B) and total protein, n = 5–6, (C) 3days after bleomycin instillation. Bars: mean±SD. **P<0.01 vs Con group; ^#^P<0.05, ^##^P<0.01 vs BLM group. The experiment was repeated three times in the same condition.

Excessive cytokine played a fundamental role in mediating the pathogenesis of ALI. The levels of IL-1β, TNFα mRNA and protein expression were significantly increased 7days after bleomycin exposure compared to that of the normal control mice in lungs(Fig 3, p<0.01). In contrast, after memantine treatment the mRNA expressions of both IL-1β and TNFα were significantly reduced compared to that of the BLM group (Fig 3, p<0.01). In addition, there was a significant alteration in IL-10 gene expression. In bleomycin injured mice, there was a dramatic decrease in lung of IL-10 mRNA level compared to that of the normal control mice (Fig 3, p<0.01). In contrast, in memantine treated mice, the IL-10 level is higher than those of BLM group (Fig 3A, p<0.05). Memantine treatment also increased the reduction in IL-10 protein expression by 24% compared to that of BLM group, although this trend was not significant (Fig 3B). These data indicated memantine protected lung from bleomycin injury by reducing the pro-inflammatory IL-1β and TNFα and elevating the anti-inflammatory IL-10.

### Effects of memantine on pulmonary permeability in lungs

**Fig 4:**
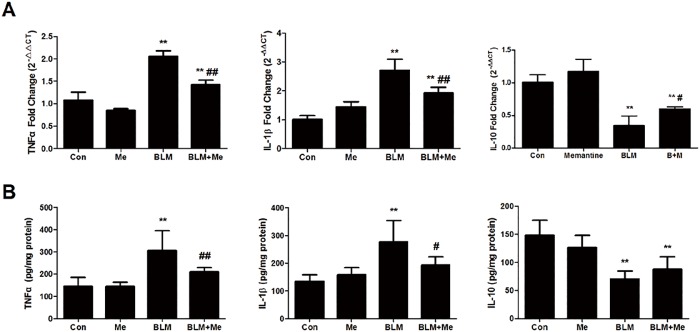
Memantine changed BLM induced gene expression of cytokines in lungs. The lung tissues were collected on Day 7. The levels of TNFα, IL-1β and IL-10 in lung homogenate were quantified by RT-qPCR (A) and ELISA (B). n = 4–6. Bars: mean ±SD. **P<0.01 vs Control group; ^#^P<0.05, ^##^P<0.01 vs BLM group.

Evans Blue dye is a molecular compound, which combines with albumin in the circulation. The combined Evans Blue dye should not cross through vascular barrier under normal conditions. The increased concentration of Evans Blue in BALF indicates the increased permeability of the lung air-blood barrier. BLM treatment results in a significant increase in Evans Blue and total protein in BALF as compared to that of the control group (Fig 4). In contrast, memantine treatment prevented BLM-induced Evans Blue and total protein increase in BALF (Fig 4A and 4C). In addition, BLM also promoted acute cellular inflammation as demonstrated by an increase of total inflammatory cells in BALF. Whereas, the number of total cells (Fig 4B) and neutrophils were significantly reduced (Fig 2D) in the BALF of BLM+Me group. In addition, the lung wet-to-dry weight ratios of the BLM+Me group were significantly decreased than those of the BLM group (Fig 2C). Our findings strongly support the protective effect of memantine against BLM-induced pulmonary permeability and barrier dysfunction.

### Effects of Memantine on MDA Contents

**Fig 5:**
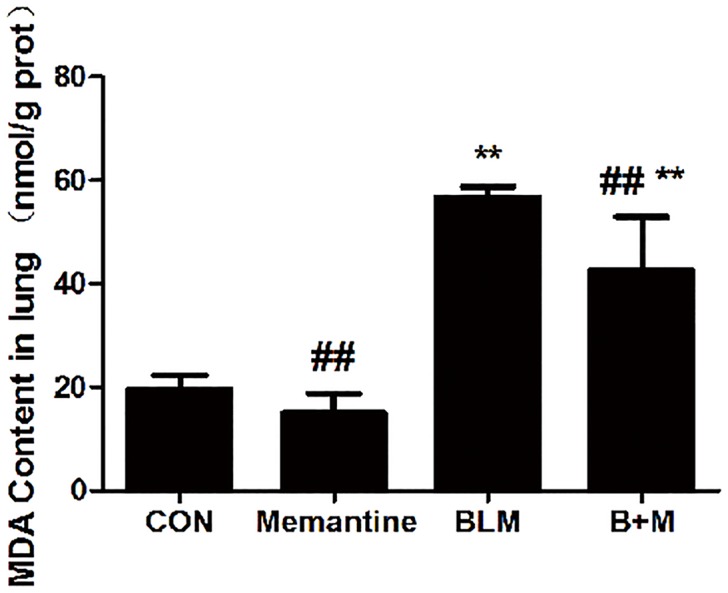
Effect of memantine on bleomycin-induced increases in the tissue malondialdehyde (MDA) levels. The lung tissues were collected on Day 7. n = 3–6. Bars: mean±SD. **P<0.01 vs Con group; ^##^P<0.01 vs BLM group. The experiment was repeated three times in the same condition.

Oxidative stress has been proved to be an important factor in the development of BLM-induced pulmonary toxicity [32]. Bleomycin treatment resulted in a significant rise in lung tissue MDA, which is an index for lipid peroxidation, when compared with the control saline group. Memantine clearly prevented the increase of MDA contents in the bleomycin stimulated lung tissue (Fig 5). This indicated that memantine had a protective role against BLM-induced injury by inhibiting oxidative stress.

### CD11b expression on neutrophils induced by NMDA in vitro

**Fig 6:**
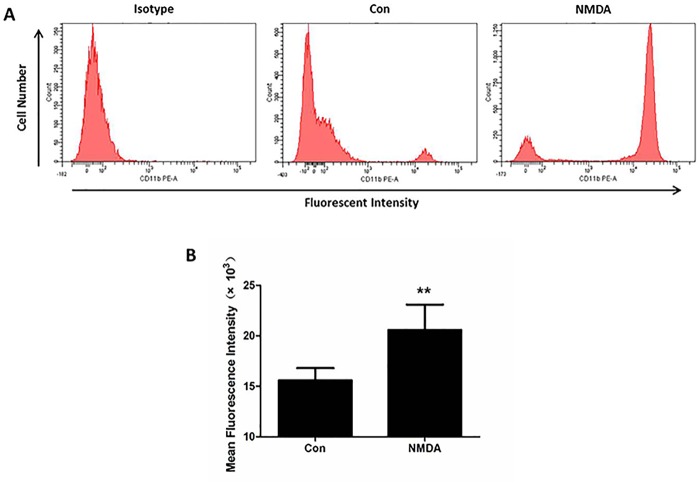
NMDA enhances the expression of CD11b on human neutrophils in vitro. (A) Representative flow cytometry plots detecting CD11b on neutrophils at 4h after normal saline or 10^-6^ mol/L NMDA exposure are shown. (B) Mean fluorescence intensity was measured to evaluate the expression of CD11b on neutrophils in different groups. n = 3. Bars: mean ±SD. *P<0.05 vs Con group. The experiment was repeated three times in the same condition.

The process of neutrophils adhering to endothelial cells is partially dependent on the interaction between CD11/CD18 on the neutrophil and ICAM-1 on endothelial cells [33, 34]. To find the effect of NMDAR on promoting neutrophilic exudate, we observed the change in CD11b expression on neutrophils after NMDA treatment in vitro. Fig 6A is a representative flow cytometry plot detecting CD11b on neutrophils at 4 h after saline or NMDA (10^-6^ M) exposure. FACS analysis showed that the mean fluorescent intensity per 10,000 cell sample was increased by 30% in NMDA group compared to the control group (Fig 6B, P<0.05). These results demonstrated that activation of neutrophil NMDAR enhanced the expression of the neutrophil adhesion molecule invoking firm adhesion and extravasation.

## Discussion

In the present study, we showed that bleomycin challenge induced selective release of endogenous glutamate in lungs and the ability of memantine, a well-known NMDAR antagonist, to mitigate bleomycin-induced lung inflammation. Specifically, mice treated with memantine have significantly less histology damage, wet/dry ratio and pulmonary permeability. Mechanistically, memantine treatment appears to mitigate lung damage by suppressing neutrophils accumulation, decreasing IL-1β, TNFα and increasing IL-10. Furthermore, our results showed that activated NMDAR increased the expression of adhesion molecule CD11b on neutrophils. Thus, our findings demonstrate that memantine protected mice from the development of bleomycin-induced acute pulmonary inflammation.

NMDARs are ionotropic glutamate receptors. In CNS, overstimulation of NMDAR leads to excitotoxic neuronal cell death under many conditions, including ischemic stroke, traumatic brain injury, Alzheimer’s disease, Parkinson’s disease, and epilepsy [9, 35]. It requires two agonists, glutamate and glycine, to activate and open NMDAR [36]. In addition, NMDAR activation induces neuroinflammation. It was pointed out that NMDA could induce retinal damage, through up-regulation of pro-inflammatory cytokines, such as IL-1β and TNFα, inflammatory adhesion molecules, and recruitment of leukocytes into the retina [15, 37]. It’s worth stressing that glutamate was released by astrocyte and activated microglia during neuroinflammation [38]. In the CNS, both in vivo and in vitro, previous studies indicated that the increase in pro-inflammatory cytokines can be reduced by NMDAR antagonist MK-801 in morphine-tolerant rats [19] and by NMDAR1 siRNA in parkinsonian cell models [39]. Memantine, a well characterized NMDAR antagonist, is used to treat dementia and Parkinson’s disease in clinic [40], and it also protected neurons from some models of neurological injury such as traumatic brain injury, ischemic stroke, spinal cord ischemia or neuroinflammation [41, 42]. Therefore, in the process of neuroinflammation, the release of Glu and the activation of NMDAR can enhance neuroinflammation and further deteriorate the CNS injury.

BLM induces lung injury via causing DNA strand breakage and oxidant injury [2]. In the early stage of BLM-induced lung injury, the inflammatory response dominates, with induction of pro-inflammatory cytokines and accumulation of monocytes. For the first time, this study showed that there was a great release of glutamate in bleomycin-induced lung injury and NMDAR antagonist memantine attenuate BLM-induced acute lung injury. This result suggested that glutamate play an important role in bleomycin-induced lung injury. In other acute lung injuries, such as hyperoxia-induced lung injury, we also found that the concentration of glutamate in BALF increased [22], and that NMDAR antagonist MK-801 can prevent the injury induced by hyperoxia [22] and experimental sepsis [28, 30]. These suggested that Glu, as an endogenous injury factor involved in different lung injury. But the cellular source of Glu needs further investigation.

Recently, NMDARs were detected in the lungs [9]. Activation of NMDARs in rat lungs triggers an acute increase in wet-to-dry lung weight ratio and protein leakage into the alveolar space, leading to pulmonary edema [20]. The mechanisms of acute lung injury induced by activation of NMDAR are not completely understood. Based on the isolated perfused and ventilated rat lung, Said *et al*. reported that lung injury caused by the NMDA in the perfusate was NMDAR mediated and NO dependent, and was associated with the increased production of NO [20]. Our previous study demonstrated that NMDA could increase NO production and nitric oxide synthase (iNOS) activity in rat alveolar macrophage [14]. NO interacts with O_2_•– to form ONOO•–, which can cause lipid peroxidation [43]. In the CNS, overactivation of NMDAR contributes to the production of ROS in neurodegenerative diseases [44]. It has been shown recently that acute kidney injury is associated with the activation of NMDA receptor induced aggravated oxidative stress [45]. Said reported that NMDA receptor blocker MK-801 attenuated oxidative acute lung injury in paraquat or xanthine oxidase models [46]. They concluded NMDAR activation plays a critical role in oxidant tissue injury [46]. Lipid peroxidation is one of the key mechanisms by which ROS induces cell death [43]. MDA is used as a marker of lipid peroxidation. The increase of MDA in the lung tissue indicates the oxidative injury. We observed increase of NOS and XOD activity and MDA, NO levels in ALI lung induced by intraperitoneally injected Glu [21]. In the present research, we found that memantine had a protective role against BLM-induced toxicity by inhibiting lipid peroxidation. It is suggested that NMDAR activation enhanced oxidative lung injury in the process of BLM-induced acute lung injury.

Our previous study demonstrated that the administration of glutamate or NMDA via intraperitoneal injection provoked acute lung injury, including increase of W/D radio and neutrophils emigration [21, 47]. Blocking of NMDAR by memantine not only decreased pro-inflammatory cytokines IL-1β and TNFα, but also increased anti-inflammatory cytokine IL-10 in the present study. These pro-inflammatory cytokines, such as IL-1β and TNFα, play important roles in leukocytes recruitment and lung injury. IL-1β and TNFα exacerbate inflammation, cause damage, and recruit neutrophils to lung in acute lung injury. IL-10, a potent anti-inflammatory cytokine, inhibits activation of macrophages and their cytokine production. The results indicated that pro-inflammatory cytokines decrease and anti-inflammatory cytokines increase are an important mechanism by which memantine carries out its protective effect on BLM-induced acute lung injury.

Neutrophils adhering to pulmonary capillaries endothelium is the first step for neutrophilic exudation, after which they migrate into alveolar spaces[48]. It was reported that MK-801, an antagonist of NMDA receptor, blocks hypoxic blood-brain-barrier destruction and leukocyte adhesion [49]. In addition, pulmonary vascular permeability was increased by administration of the NMDAR agonist glutamate a process inhibited by MK-801 [50]. In the present study, we found that memantine can prevent neutrophils recruitment in the BLM-injured lung and decrease the permeability of lung capillary-alveolar barrier. This indicates that inhibition of neutrophils emigration into the lung may be another mechanism of memantine attenuating BLM-induced lung injury. Interactions between CD11b/CD18 on neutrophils and ICAM-1 on endothelial cells are important for neutrophils adhering to endothelial cells [33]. CD11b/CD18 are important in the adhesion of neutrophils [34]. Our results showed that NMDA treatment could increase the expression of adhesion molecule CD11b on neutrophils. Others showed that NMDA increased endothelial adhesion molecules, including ICAM-1 in the retinal vessels [37]. NMDA receptor blockade abolished MgD-related increase of monocyte surface protein CD11b expression [17]. This evidence suggests that NMDAR activation promotes neutrophils emigration into the lung by strengthening adhesion between neutrophils and endothelial cells.

In summary, findings of our study showed for the first time that memantine could attenuate bleomycin-induced lung inflammation. However, further studies are required to evaluate the role of memantine in the prevention and the treatment of lung fibrosis. In the present study, memantine holds promise as a novel drug to treat bleomycin-induced lung inflammation.
